# The Immunological Role of Milk Fat Globule Membrane

**DOI:** 10.3390/nu14214574

**Published:** 2022-10-31

**Authors:** Maria Cavaletto, Annalisa Givonetti, Chiara Cattaneo

**Affiliations:** 1 Department of Sviluppo Sostenibile e la Transizione Ecologica, University of Piemonte Orientale, 13100 Vercelli, Italy; 2 Department of Scienze e Innovazione Tecnologica, University of Piemonte Orientale, 15121 Alessandria, Italy

**Keywords:** human milk, milk fat globule membrane, MFGM, breastfeeding, immunometabolism

## Abstract

Human milk is the ideal food for newborns until the age of six months. Human milk can be defined as a dynamic living tissue, containing immunological molecules, such as immunoglobulins, supra-molecular structures, such as the milk fat globule membrane (MFGM), and even entire cells, such as the milk microbiota. The milk composition changes throughout lactation to fulfill the infant’s requirements and reflect the healthy/disease status of the lactating mother. Many bioactive milk components are either soluble or bound to the MFGM. In this work, we focus on the peculiar role of the MFGM components, from their structural organization in fat globules to their route into the gastrointestinal tract. Immunometabolic differences between human and bovine MFGM components are reported and the advantages of supplementing infant formula with the MFGM are highlighted.

## 1. Introduction

Breastfeeding is the source choice for infant nutrition; breastfeeding is recommended during the first 6 months of life [1]. Human milk contains many bioactive components, ideally shaped for newborn development, both from a nutritional and immunological point of view. Clinical trials have shown that breastfeeding lowers the risk of intestinal and respiratory tract infections [2]. Recently, one prime area of research in the field of breastfeeding and human milk involved improving the awareness of evidence-based benefits and challenges of breastfeeding among health practitioners and the public [3]. Human milk contains immune-active molecules, such as immunoglobulins, cytokines, membrane, and soluble proteins with immunological properties and immune cells; together, these components protect infants against infectious agents and drive infant tissue development. One of the most important bioactive components of milk is the milk fat globule membrane (MFGM) [4,5]. This component consists of a tri-layer membrane containing proteins (many of them glycosylated), cholesterol, and polar lipids, among them, phospholipids, sphingolipids, and gangliosides. MFGM, due to its complex architecture, is highly resistant to digestive enzymes and can display its immunological action directly in the small intestine of the infant. Here, the intestinal barrier epithelium provides a first defense against infections by engaging innate and adaptive mucosal immune components; MFGM and human milk microbiota actively cooperate with the intestinal barrier to establish healthy microbiota in infants [6].

Moreover, the MFGM components have a role in directing the metabolisms of infants. The infant brain requires approximately 74% of the total dietary energy intake, whereas in adults, only 20–23% of dietary energy is used to feed the brain. Human newborns have higher brain–body mass ratios and have more body fat than babies of other mammals. Several studies support the benefits of breastfeeding in improving cognitive development and preventing children from becoming overweight or obese in their young adult years [7].

In the intestinal tract, human MFGM and the other milk components fuel the cells and drive their metabolic pathways. It has been shown that metabolic pathways are pivotal during immune cell activation, either in proinflammatory or in anti-inflammatory responses [8,9]. The central metabolism processes of each cell are fundamental for nutrition, as well as regulating inflammation and the host’s response to infection. A newborn’s reactivity to infections relies mostly on innate immune mechanisms. Interactions between metabolism and the immune response are increasingly recognized; changes in the cellular metabolic status are able to push innate immune cell activation and, consequently, host responses to infections [10,11].

## 2. Composition of MFGM

The human milk composition is always in transition; it can be defined as dynamic living tissue [12]. Milk contains lipids, proteins, carbohydrates, enzymes, hormones, cytokines, vitamins, minerals, immunoglobulins and microRNAs, and human and microbial cells. Milk’s composition is approximately 87% water, 7% lactose, 3.8% lipids, and 1.0% protein. Lactose is the main carbohydrate present in human milk and is responsible for 40% of the total energy, followed by oligosaccharides, which have an important role in gut health. The lipid fraction, represented by triacylglycerols, is responsible for about 50% of the energy provided by milk. Triacylglycerols are secreted as fat globules in the mammary gland, enveloped by the MFGM, which is a tri-layer structure of polar lipids and membrane-associated proteins [5,12]. The most abundant polar lipids in the human MFGM are sphingomyelin, 27–43%, followed by phosphatidylcholine 14–38%, and phosphatidylethanolamine 6–36%; phosphatidylserine and phosphatidylinositol are less abundant, at around 5–6%. Gangliosides are present in trace amounts [13]. The latter are resistant to digestion and play an important role in the gut–brain axis and in the development of the neonatal immune system [14]. As described in detail [15], the composition of MFGM in polar lipids varies throughout lactation, from colostrum to mature milk, and even by the geographical areas of the mothers. MFGM polar lipids, such as phospholipids, gangliosides, and sphingomyelin, have been shown to exert beneficial effects on intestinal health [16]. The lipid portion of MFGM is organized into two distinct lipid domains. Sphingomyelin is associated with cholesterol and forms a densely packed phase. This sphingomyelin-rich domain or raft domain is encompassed by a less densely packed matrix composed of glycerophospholipids [17]. The composition of the fatty acid profile has revealed that in MFGM, there is a higher amount of saturated fatty acids than in the total human milk fat for all lactation phases [18]. This feature is reflected in the MFGM microstructure, influencing the degree of fluidity, and it could have a role during the digestion and interaction with other molecules in the gut lumen. Several studies have suggested that supplementing infant formulas with MFGM could provide beneficial effects because of the presence of bioactive compounds, such as proteins and polar lipids in the MFGM, thus reducing the gap between human milk and infant formulas [16,19].

Advanced and comparatively proteomic investigations elucidated the different and conserved protein compositions of MFGM from the milk of different species [20]. Major MFGM proteins are mucin I, xanthine oxidase, lactadherin, cluster of differentiation (CD) 36, butyrophilin, adipophilin, and fatty acid binding protein. Their protective functional properties are well established and annotated in UniProt [21]. Among minor proteins associated with MFGM, the presence of toll-like receptors (TLRs) is noteworthy. TLR expression is higher in human milk compared to other milks. TLR2 is extremely important for immediate and efficient recognition of bacterial lipoproteins and cell wall components during innate immune response [22].

## 3. Immuno-Metabolism of MFGM

MFGM is a small but—in terms of biological activities—significant fraction [23]. The metabolic route of MFGM through the gastrointestinal tract is depicted. There are significant differences in the physiology of digestive process between infants and adults because of the immaturity of the gastrointestinal tract at birth; nevertheless, milk digestion in infants is a sequential and balanced process [17]. Gut morphology and immunometabolic function development are fostered by constant interactions among dietary components, host gastrointestinal cell activity, and microorganisms. Moreover, the interplay between the microbiota and gut immunity determines the level of inflammation.

In the first months of life, the dietary protein needs of the infant drop rapidly; consequently, protein concentration in human milk declines as lactation proceeds toward the secretion of mature milk. Unfortunately, infant formula composition is not adjusted to follow the time-dependent changes in protein requirements and its total protein concentration is higher than human milk. Recently, a clinical trial investigated the differences between the breast-fed and formula-fed infant serum metabolome, an elevated level of ketogenesis was highlighted in breast-fed infants, with the detection of increased 3-hydroxybutyrate, acetate, and formate (the latter as by-products of lipid oxidation). In contrast, the serum metabolome of formula-fed infants showed the prevalence of protein catabolism, showing metabolites, such as urea, essential amino acids, and amino acid catabolism by-products [7]. From a metabolic perspective, supplementation of infant formula with MFGM altered the metabolic performances of the newborns, with a marked shift toward a preference for fat utilization (typical of breast-fed infants), from a preference for protein utilization (typical of formula-fed infants), and changed the gut microbiota to become more similar to that of breast-fed infants [7,24]. The high levels of ketone bodies found in the blood of a breast-fed infant are able to sustain the rapid growth and development of the infant’s brain. In fact, glucose metabolism in the brain of the breast-fed infant is lower than in the adult state; during this period, the brain can efficiently use ketone bodies. Ketone bodies provide alternative sources of fuel for the brain, and serve as building blocks for endogenous synthesis of cholesterol, long chain fatty acids, and amino acids that are important for the developing brain. Furthermore, a signaling role for 3-hydroxybutyrate at the cellular level has been recognized [25].

The high sensitivity to lipolysis, lipid-oxidation, and ketogenesis in early life can inhibit excessive weight gain in later life and, thus, decrease the risk of obesity. Thus, at the moment, supplementing infant formula with a bovine MFGM preparation may be the best and most sustainable choice to address the nutritional gaps observed between breast-fed and formula-fed infants [26].

### 3.1. Gastric Environment

The high level of glycosylation of the MFGM proteins protects them from degradation by pepsin and denaturation by the low pH of the infant’s stomach [27]; the result is that MFGM proteins maintain their functional bioactivity in the gut. As for MFGM lipids, their digestion starts in the infant’s stomach; as the pH falls below 5.5, the MFGM structure becomes less stable, and leads to coagulation of the fat globules [16] Gastric lipase is highly expressed in the neonate; it is resistant to pepsin and can be adsorbed at the interface of fat globules and, thus, initiate fat hydrolysis [28].

### 3.2. Small and Large Intestines

In the infant’s intestine, differing from an adult’s, during fat digestion, the main roles involve bile salt-stimulated lipase (BSSL) and pancreatic lipase-related protein 2 (PLRP2); these two enzymes compensate for the low activity of pancreatic triacylglycerol lipase and the small amount of bile salts [17]. The products of gastric lipase, 1,2-diacylglycerols are further digested by PLRP2 and BSSL. Moreover, triacylglycerols, diacylglycerols, and phospholipids are hydrolyzed by BSSL, which is highly active during the neonatal period and is typical of human milk; it has not been detected in bovine milk [17]. Dietary sphingomyelin is hydrolyzed in the intestinal mucosa by alkaline sphingomyelinase to produce phosphocholine and ceramide. In a subsequent step, neutral ceramidase releases sphingosine from ceramide; sphingosine is adsorbed, phosphorylated to sphingosine-1-phosphate, and converted to palmitic acid in the intestinal epithelial cells [29]. The MFGM structure may be important in the route through the infant gut, it allows the delivery of sphingosine and ceramides directly to the distal gut. The overall rate of fatty acid release in infant formula is significantly reduced, while that in human milk remains relatively high during the second hour of intestinal digestion; a similar trend is evaluable for gastric digestion [28,30]. Orally ingested MFGM increases intestinal differentiation and the expression of tight junction proteins in rat pups and mediates permeability in a human enterocytes model [31].

Since glycosyl moiety protects MFGM proteins from cleavage by gastrointestinal proteases, they can display their bioactivity in the intestine. Mucin and lactadherin contribute to the infant’s health and development by displaying antiviral and antibacterial activities in the lumen of the gastrointestinal tract; butyrophilins have been related to the modulation of the immune response; xanthine oxidase exerts an antimicrobial effect due to its role in the production of reactive oxygen species. Furthermore, a certain degree of bioactivity is shown by the peptides originating from the partial digestion of these main MFGM proteins (mucin, lactadherin, and butyrophilin), considering the higher permeability of the infant’s intestinal tract [16].

In an animal model, it has been shown that after transit in the small intestine, bovine MFGM proteins and peptides appeared to be almost fully hydrolyzed by the intestinal proteases, regardless of the MFGM preparation the rats received; with the exception of mucin 1, which has been shown to have a partial resistance to intestinal degradation [32].

### 3.3. Interaction with the Microbiota

Gut microbiota, including a possible fetal microbiota, provide the infant with both genetic and metabolic agents, and perform the synthesis of various inaccessible nutrients, such as amino acid derivatives, vitamins, and short-chain fatty acids, and improve the enzymatic capacity of the intestine (providing the necessary enzymes to digest complex carbohydrates). The origin of a fetal microbiome is still highly questioned [33,34]. The development of infant microbiota is influenced by different factors: the maternal use of drugs (antibiotics), if the infant is born premature or at term, the mode of delivery, and the infant’s diet (composition of the mother’s milk, donor milk, formula milk). The proliferation of stem cells by microbial metabolites and the infant’s immune-metabolic development are made easier by a more diverse infant gut microbiota at birth [33]. Microbiota provide protection against pathogens, stimulate innate and adaptive (humoral and cellular) immune responses, and regulate the development of enterocytes [35].

It has been shown that antimicrobial peptides and surface glycosyl moieties of milk fat globules (MFG) could have an important role in shaping gut microbiota [30]. On the other hand, gut microbiota can also contribute to milk fat digestion by modulating the expression of lipid metabolism enzymes. It has been demonstrated that the microbiota of the small intestine tunes the host’s adaptation in response to dietary lipids; this synergism could be exploited in the preparation of infant formula with probiotics and MFGM to improve lipid digestion [35].

One of the major risks for developing necrotizing enterocolitis and respiratory tract infections is caused by a high permeable intestinal barrier, or “leaky gut”, a common condition in preterm newborns [36,37,38,39]. During the first week of life, the most rapid postnatal intestinal maturation occurs; with the use of high-resolution sequencing approaches, it has been possible to characterize the composition of the developing gut microbiota in the first week of life. Thus, *Bifidobacterium* species were identified as a significant biomarker of postnatal intestinal barrier maturation [40]. Supplementation of MFGM in early life can modulate the gut microbiota and, consequently, the gut–brain axis, and provide better cognitive development [41,42]. Leaky gut and enteric dysfunction are prevalent in children living in unsanitary conditions; nevertheless, they are also in high-income countries. Overnutrition and/or better ultra-processed food consumption lead to leaky gut in both the mother and the child, leading to gut inflammation, dysbiosis, bacterial translocation, systemic inflammation, and nutrient malabsorption [43].

In leaky gut, the MFGM can act as a decoy for detrimental microbiota, reducing the translocation of the microbial debris to the subepithelial environment and the amount of immune responses. Without bacterial translocation, the activation of TLR4 by bacterial lipopolysaccharides does not occur and, consequently, the pro-inflammatory cascade in immune cells is not activated [33]. Gut microbiota and leaky gut can be both affected by neurological diseases; reciprocally, intestinal disorders, such as inflammatory bowel syndrome(s) (IBS), can be responsible for the development of neurodegenerative diseases. Since Firmicutes (F) and Bacteroidetes (B) are the two main gut phyla, the F/B ratio is used to evaluate gut health. High values of F/B ratio have been correlated with obesity, aging, and in IBS patients. Another major effect of leaky gut is endotoxemia, which can also induce blood–brain barrier hyperpermeability and activate TLRs on the endothelial surface [44]. The growth of pathogenic bacteria, including *Streptococcus pneumoniae* and *Haemophilus influenzae*, has been inhibited by human milk oligosaccharides (HMOs) through their interference with the adhesion of the bacteria to epithelial cells [33]. However, other bacteria, including Bifidobacterium and Bacteroides, use HMOs for energy production in central metabolism, with the concomitant beneficial release of short-chain fatty acids. The MFGM role in the modulation of the innate immune system has been shown in a rat model, in which MFGM enhanced the mucus barrier and involved the regulation of NLRP6 inflammasome [45].

Studies are emerging on the potential modulation of gut microbiota by dietary polar lipids (phospholipids and sphingolipids), which are primarily located within the MFGM. After being digested and partially adsorbed in the small intestine, a large fraction of dietary sphingomyelin and its digestive products are able to reach the colon, where they may exert their bactericidal and gut-modulating effects. *Akkermansia muciniphila* was significantly higher in milk phospholipid-fed mice. *Akkermansia muciniphila* has been shown to have positive metabolic effects, which includes improving insulin sensitivity and protecting against metabolic endotoxemia-induced inflammation. Although preclinical findings report promising results regarding the modulation of the gut microbiota by milk polar lipids, only one human clinical trial has been conducted so far [46].

### 3.4. Formula Milk Added with Bovine MFGM, an Important Supplement

When breastfeeding is not possible, an infant formula is proposed; scientific investigations are still searching the “gold standard” infant formula. For many years, the MFGM was discarded from the preparation of formula. Generally, formulas are prepared from bovine milk. It is very important to go deeper into the function and architecture of the MFGM. It is clear that components play a pivotal role for the infant, not as single molecules, but as a whole, as an interplay among different components organized in the architecture of the MFGM. Many investigations and clinical trials have been performed to highlight the differences among breastfeeding, standard formula feeding, and formula supplemented with MFGM [23]. In a standard infant formula, fats are vegetable oils, and fat globules display different architectures: they are sub-micron sizes (0.4–0.5 µm), smaller than human MFGs, and surrounded by a dense layer of milk proteins or other non-milk-derived surfactant emulsifiers (plant lecithin and monoglyceride). This implies that lipids associated with infant formula MFG cannot be completely digested by the newborn [28]. Since gangliosides are more concentrated in human milk than in bovine milk, supplementation of infant formula with dairy gangliosides resulted in positive effects on the cognitive development in infants aged 0–6 months [30]. The effect of sphingomyelin-fortified milk on the neurocognitive development of low-birth-weight or preterm infants has been shown, in fact, dietary sphingomyelin and its metabolites are able to cross the blood–brain barrier and improve myelination [47]. As much as a concern supplementation with bovine MFGM is, the industrial production processes, inducing a different interfacial structure or aggregation state of MFG, could affect the milk fat digestion rates. The homogenization step brings milk proteins (caseins and whey proteins) to the surface of MFG, but at the same time, some important glycosylated proteins are lost during due to deconstruction of the MFGM. Furthermore, thermal processing of homogenized milk induced a lower intestinal digestion rate, when tested in vitro [48]. An optimal solution would be the production of MFGM bioactive components directly from human milk, due to their excellent tolerability and absence of side effects. Nevertheless the scale-up is very difficult and limit the availability of adequate amounts of human MFGM for carrying out preclinical–clinical research studies. Commercially available MFGM isolates, used in infant formulas, are predominantly bovine-sourced, even if dairy components isolated from milk of other species, such as transgenic cattle or microbial cultures, are used [16]. For more than forty years, scientific research has been engaged in identifying differences between the MFGM of humans and other species; the task is not finished yet. For example, the differences in isoforms and glycoforms of MFGM proteins within and/or between species may be responsible for different bindings, receptor activities, signaling, and enzyme activities [30]. Currently, the source and processing for preparing MFGM-enriched formula, such as serum or cream concentrate, are responsible for the variations in the types and amounts of polar lipids and membrane proteins, and therein, their potential bioactivities. Overall, heterogeneity in the composition of commercially available MFGM is probably the major reason for the discrepancy in the beneficial effects of MFGM supplementation in infants [49]. Proteomics and lipidomics are useful strategies to investigate the composition of MFGM, nonetheless, to unravel the immunometabolic role of MFGM, it is necessary to consider MFGM as a whole [50]; in this way, animal studies and randomized clinical trials have provided evidence of the MFGM bioactive properties, as listed in Table 1 and the references therein. Further studies could be set up to reinforce the immunometabolic role of MFGM, enrolling more different geographical areas. Table 1 lists the pre-clinical and clinical studies that have tested the supplementation of bovine MFGM. It is noteworthy that two protocols have been published, the Swedish OTIS study [51] involving the supplementation of MFGM on Nordic food, a dietary plan that is low in protein and high in fruits and fish, in order to evaluate the effect on growth, gut microbiota, and cognitive development. While the second study is a Chilean protocol ChiNuT based on evaluating the effect of a MFGM-enriched formula on healthy growth and cognitive development [52]. At the moment, the results have not been published.

In Ref. [89], the authors focused on the risk–benefit of using ingredients enriched with MFGM or milk phospholipids in infant formula; in particular, they underlined two area of exploitation: the first one is infant nutrition to ameliorate formula-fed infants features; the second area is where MFGM supplementation is encouraged in the prevention of several diseases, including cardiovascular disease, impaired bone turnover, inflammation, skin conditions, cognitive decline, and muscle loss. However, although there are remarkable and measurable benefits in MFGM-enriched infant formula, the precise mechanism that leads to these benefits remains to be elucidated. One aspect is well established: there are no reported adverse effects that would increase the health risks of using MFGM supplementation [89]. Generally the outcomes presented in clinical trials were highly heterogenous in terms of the methods used to evaluate specific outcomes and time points of the assessment [49].

## 4. Conclusions

**Figure 1:**
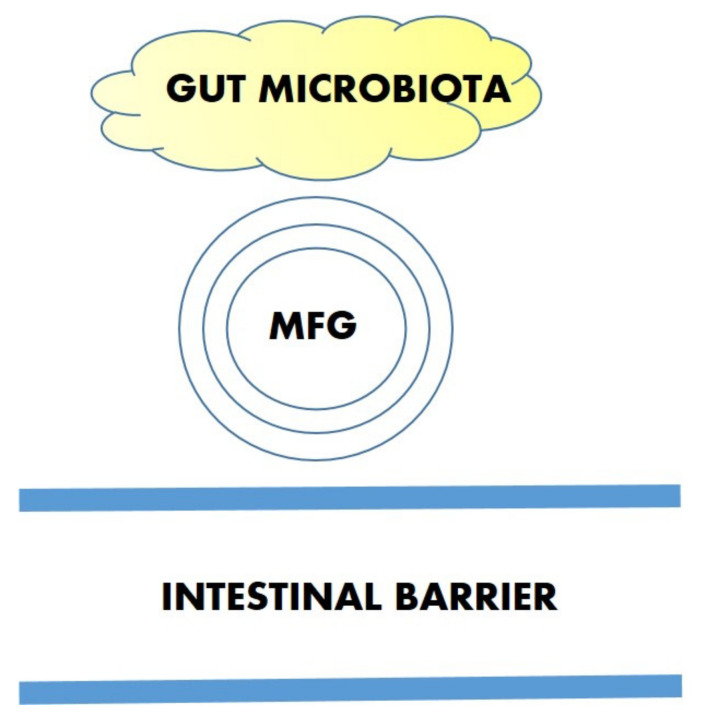
Schematic representation of the molecular–structural interplay in MFG with the tripartite membrane, intestinal barrier, and gut microbiota. This is a schematic representation; the dimensions are not in scale, but it is useful to highlight the MFGM platform role for assuring interplay among different structures.

This review highlighted an important role for human MFGM: the immunometabolic activity of its components. Taking into account the strong correlation between structure and function in biology, the peculiar organization of human MFGM is responsible for energy acquisition, immune-metabolism, and gut maturation of the breast-fed infant. The molecular mechanism, which is responsible for the bioactivity of many MFGM components, has to be fully elucidated. For future investigations, clinical trials need to follow infant cohorts beyond the first few years of life. It is important to consider that the specific interplay between the dyad mother–child cannot be replaced by an infant formula, even if it is a MFGM supplemented infant formula. The composition of human milk varies throughout lactation (even during each day) in order to satisfy the specific needs of the child. The complex bioactive role of human milk, mainly represented by the immune-metabolic role of MFGM, is further complicated by its interaction with the gut microbiota, which is different and specific for each infant (Figure 1). All of these features together can explain how it is difficult to place clinical trials for infants in the first months of their lives, and how it becomes more difficult to place follow-up actions in their childhoods and adult lives. Breastfeeding must be encouraged at medical and social levels. Donor milk or infant formula should be limited; nevertheless, scientific research efforts must unravel the interplay between human milk and the infant, in order to prepare the best infant formula approaching the “magic-living” food, such as human milk.

## Figures and Tables

**Table 1:** Studies with bovine MFGM-enriched formula or lipids as dietary supplementation.

Type of Study/Year	MFGM Formulation	Health Effects	Reference
RCT/2011 e 2018	MFGM-fortified complementary food	Lower incidence of bloody diarrhea Abnormalities removal in protein and energy metabolism, toward an enhanced immunity, decrease of T helper type 1 response, decrease of trimethylamine-N-oxide (Peruvian infants)	[53,54]
RCT/2012	Bovine gangliosides enriched formula	Better cognitive function, similar to breastfed controls (Indonesian infants)	[55]
RCT/2012	MFGM-enriched formula	Lower febrile episodes (Belgian preschool children)	[56]
RCT/2014	Bovine ganglioside enriched formula	No difference in diarrhea incidence (Indian infants)	[57]
RCT/2014 e 2015	MFGM-enriched formula	Positive effect on cognitive scores; lower incidence of otitis; higher serum cholesterol (Swedish infants)	[23,58,59]
RCT/2014	Formula enriched with a protein rich MFGM and Formula enriched with a lipid rich MFGM	For both formula no weight gain; some atopic dermatitis in protein rich MFGM group (French and Italian infants)	[60]
RCT/2017	MFGM-enriched formula	Moderate effect on oral microbiome, *Moraxella catarrhalis* was less prevalent (Swedish infants)	[61]
RCT/2018	MFGM-enriched formula	Differences in the serum/plasma lipidome at 4 and 6 months of age (Swedish infants)	[62]
RCT/2019, 2019	MFGM-enriched formula	Enriched-formula infants are more metabolically similar to BF infants. Limited differences in fecal microbiome and metabolome between Enriched-formula fed and standard formula fed infants (Swedish infants)	[7,63]
RCT/2019, 2021	MFGM plus lactoferrin enriched formula	Accelerated neurodevelopmental profile and fewer diarrhea and respiratory-associated adverse events. Increased prevalence of *Bacteroides* species n gut microbiome, modest effect on stool metabolome (Chinese infants)	[64,65]
RCT/2019, 2021	MFGM- or probiotic enriched formula	Fewer fever episodes compared to standard formula, the outcomes of MFGM group were close to those of the breastfed group, Cytokine profile of the MFGM group approached that of breastfed infants, (Chinese infants)	[66,67]
RCT/2021	MFGM-enriched formula	Serum gangliosides increase and improvement of cognitive development (Chinese infants)	[68]
RCT/2021	MFGM plus iron enriched formula	Well-tolerated, adequate growth, normal iron status until one year of age (US infants)	[69]
RCT/2021	MFGM-enriched formula	Serum metabolome shifts to fatty acid oxidation and ketogenesis (Chinese infants)	[26]
RCT/2022	MFGM-enriched formula	The MFGM-formula had a similar digestive tolerance to the standard formula and was different from the breastfeeding in stool color (Chinese infants)	[70]
RCT/2022	MFGM-enriched formula	Supported growth of *Bifidobacterium* in gut microbiome (Chinese infants, limited number)	[71]
RCT/2022	MFGM plus PUFA plus symbiotic-enriched formula	Neurocognitive positive effect. Long term effect at 6 years of age of supplementation in the first months of life. Bifidogenic and lactogenic effect on gut microbiota (Spanish infants)	[72]
Animal study/2016	Supplement of MFGM plus prebiotic plus lactoferrin	Advance in neurodevelopment (piglets)	[73]
Animal study/2017	MFGM-enriched formula	Protection against inflammation, development of intestinal barrier and microbiome (rats)	[74]
Animal study/2018	Mixture of vegetable oils plus milk fat plus MFGM	Developmental profile of intestinal physiology and mucosal immunity were positively modified (piglets)	[75]
Animal study/2018	MFGM-enriched formula	Promotion of reflex development, change in brain phospholipid composition (rats)	[76]
Animal study/2018	MFGM addition to high fat diet	Protection against diet-induced adiposity by suppressing adipogenesis and promoting brown-like transformation of white adipose tissue (mice)	[77]
Animal study/2020	MFGM-supplemented diet in late gestation	Positive effects on intestinal barrier (neonatal piglets), improvement of plasma parameters and gut microbiota (sows)	[78]
Animal study/2020, 2021	MFGM plus polar lipids supplementation in basal and high fat diet	Maternal treatment in high fat diet reduced adiposity and induced thermogenesis in offspring. Neurodevelopment and alleviation of cognitive impairment in the offspring (pregnant rats and their offspring)	[79,80]
Animal study/2020	MFGM plus prebiotic blend	Modification of the gut microbiota, improvements of the long-term effects of early-life stress (maternal separation in rats)	[81]
Animal study/2020	Intragastrically administered formula with MFGM	Positive effects on intestinal physiology and gut microbiota (rats)	[82]
Animal study/2021	MFGM supplementation	Modulation of gut microbiota and protective effect on glucose and lipid metabolism (mice offspring exposed to maternal high fat diet)	[83]
Animal study/2021, 2022	MFGM supplementation	Improvement of the colonic mucus barrier and regulation of NLRP6 inflammasome; Positive effect on liver injury by inhibiting the activity of the autophagy-inflammasome (rats with short bowel syndrome)	[45,84]
Animal study/2022	Oral supplementation of MFGM	Intestinal barrier differentiation, increase expression of tight junctions proteins (rats)	[31]
Animal study/2022	MFGM-enriched formula	Modification of the hippocampus lipidome (piglets)	[85]
Animal study/2022	MFGM supplementation in basal and high fat diet	Neuroinflammation reduction by decrease of lipopolysaccharides and pro-inflammatory cytokines in the circulation and brain, inhibition the activation of microglia, downregulation of pro-inflammatory bacteria in the gut (obese rats)	[86]
Animal study/2022	MFGM supplementation	Reduction of colitis and hepatic injury, positive effect on the mucosal barrier and bacterial community, oxidative stress inhibition (mice)	[87]
Animal study/2022	MFGM-enriched formula	reduction of induced visceral hypersensitivity, better cognitive performance (maternal separation in rats)	[88]

RCT: randomized controlled trial; PUFA polyunsaturated fatty acids.

## Data Availability

Not applicable.
